# How Does Anxiety and Depression Affect the Outcome after Periradicular Infiltration Therapy?—A Retrospective Analysis of Patients Undergoing CT-Guided Single-Level Nerve Root Infiltration Due to Chronic Monoradicular Pain

**DOI:** 10.3390/diagnostics13182882

**Published:** 2023-09-08

**Authors:** Chris Lindemann, Alexander Hölzl, Sabrina Böhle, Timo Zippelius, Patrick Strube

**Affiliations:** 1 Orthopedic Department, Jena University Hospital, Campus Eisenberg, Friedrich Schiller University, 07607 Eisenberg, Germany; a.hoelzl@waldkliniken-eisenberg.de (A.H.); s.boehle@waldkliniken-eisenberg.de (S.B.); timo.zippelius@rku.de (P.S.); 2 Department of Orthopedic Surgery, University of Ulm, 89081 Ulm, Germany; p.strube@waldkliniken-eisenberg.de

**Keywords:** depression, anxiety, lumbar spine, chronic low back pain, radiculopathy, periradicular infiltration

## Abstract

The aim of this study was to research the influence of psychological confounders on patient-reported outcomes (PROs) after lumbar infiltration therapies of periradicular infiltrations (PRI). Patients who underwent PRI in a single center between June 2018 and December 2019 were included. PRI was performed in patients with predominantly unilateral lumbar radiculopathy which existed for at least 6 weeks based on single-level nerve root compression (caused by a herniated disc, stenosis of the lateral recess, or neuroforamen), confirmed by morphological imaging. The numeric pain rating scale (NRS) for back pain (BP) and leg pain (LP) and the Oswestry Disability Index (ODI) were assessed preinterventionally, on the first day (only NRS) and at 6 weeks, and then 3, 6, and 12 months postinterventionally. The minimally clinically important difference (MCID) served as the threshold for the therapeutic effectiveness evaluation. The health-related quality of life (SF-36) was recorded preinterventionally and after 12 months. Based on the Hospital Anxiety and Depression Scale, the patients were dichotomized into depressed or nondepressed and anxious or nonanxious. Categorical data were evaluated using Fisher’s exact test, and continuous data were evaluated using Student’s *t* test. Separate linear mixed models were built to estimate the effect of anxiety or depression on repeatedly measured PROs following PRI. Data were analyzed using SPSS software. The analysis included 102 patients. Most mean baseline PROs were significantly worse in anxious or depressed patients than in nonanxious or nondepressed patients: Anxiety NRS-BP (*p* = 0.007), ODI (*p* < 0.001); Depression NRS-BP (*p* = 0.026), NRS-LP (*p* < 0.001), ODI (*p* < 0.001). All patients showed a clinically meaningful reduction in pain and functional improvement over a 12-month follow-up. There was no significant difference in the estimated overall mean PRO between all patients (*p* > 0.05). In conclusion, anxiety and depression are associated with worse PROs before and after PRI. However, patients with underlying depression or anxiety can expect a similar gain in PRO compared to patients without depressive or anxious symptoms.

## 1. Introduction

Degenerative changes in the lumbar spine are a common cause of chronic back pain (LBP) and radiculopathic pain (RP), physical limitations, and a reduced health-related quality of life. Moreover, they are associated with significant social and health care costs in Western societies and have a growing lifetime prevalence [1,2,3]. The treatment of LBP and RP is, therefore, particularly important. Only a small proportion of LBP and RP patients undergo surgery [4]. Consequently, nonoperative modalities such as physical therapy, analgesic agents, and injection therapy are a significant factor in overall costs. In particular, the implementation of infiltration therapies such periradicular injections (PRI) in patients with primarily RP has appeared to be increasing at a significant rate over the past few decades [5]. However, the efficacy of lumbar infiltration therapies is controversially discussed [6,7]. PRI responses undoubtedly depend on several factors, including disease pathology and an array of patient attributes [7,8,9]. Therefore, it is crucial to determine how individual patient characteristics, symptoms, imaging findings, and anticipated method of steroid injection influence the likelihood of a meaningful response to such lumbar infiltration therapies. Moreover, it is known that psychological comorbidities such as depression and anxiety lead to increased back pain and decreased functionality [10]. Multiple studies have also demonstrated an association between preoperative depression and poor outcomes following lumbar spine surgery [11,12,13]. However, very few studies have analyzed the relationship between psychological comorbidities (e.g., depression and anxiety) and results after lumbar infiltration therapies with a lack of long-term follow-up [14,15]. Consequently, the early identification of patients who are more likely to improve with lumbar infiltration therapy may lead to better outcomes and even reduced costs.

This study aimed to compare the outcome of patients after PRI with and without psychological confounders to support clinical decision making in this patient population. Our primary hypothesis was that patients with anxiety and/or depression before PRI would show less pain and functional improvement than patients without such confounders. Our secondary hypothesis was that patients with anxiety and/or depression have more pain and lower function before and after PRI.

## 2. Materials and Methods

### 2.1. Patients and Study Design

Patients from a database researching the diagnostic value of PRI at a university spine center between June 2018 and December 2019 were included in this retrospective, single-center, cohort study after screening for inclusion/exclusion criteria. This manuscript adheres to the applicable STROBE guidelines [16]. The inclusion criteria were patients aged ≥18 years with predominantly monoradicular leg pain after the failure of structured noninvasive conservative treatment with pain relievers and physiotherapy for at least six weeks and a complaint duration of at least 12 weeks. PRI was performed in patients with predominantly unilateral lumbar radiculopathy based on single-level nerve root compression (caused by a herniated disc, stenosis of the lateral recess, or neuroforamen) confirmed by morphological imaging (MRI or CT). The definition for predominant pain resulted from the highest NRS value (back vs. leg). All patients were mentally and physically capable of providing consent and processing the questionnaires. During follow-up, included patients were able to receive structured conservative therapy using analgesics and physical therapy.

To avoid a study bias the exclusion criteria were previous surgeries on the affected spine segment and bilateral radicular complaints, and multilevel pathologies in the MRI of the lumbar spine. Furthermore, patients with an increased peri-interventional risk profile due to other diseases were excluded: e.g., clotting disorders, insufficiently controlled diabetes mellitus, intake of oral anticoagulants, leukocytosis and increased CRP, known allergy to local anesthetics or steroids, and known infections and/or cancer diseases. Patients who had an absolute surgical indication due to acute serious neurological deficits (e.g., paresis > 3/5 according to Janda and conus/cauda syndrome) were also excluded from the study. Regarding other possible confounders in terms of treatment success, patients with known addictive disorders or proven psychological pain were also excluded.

**Figure 1:**
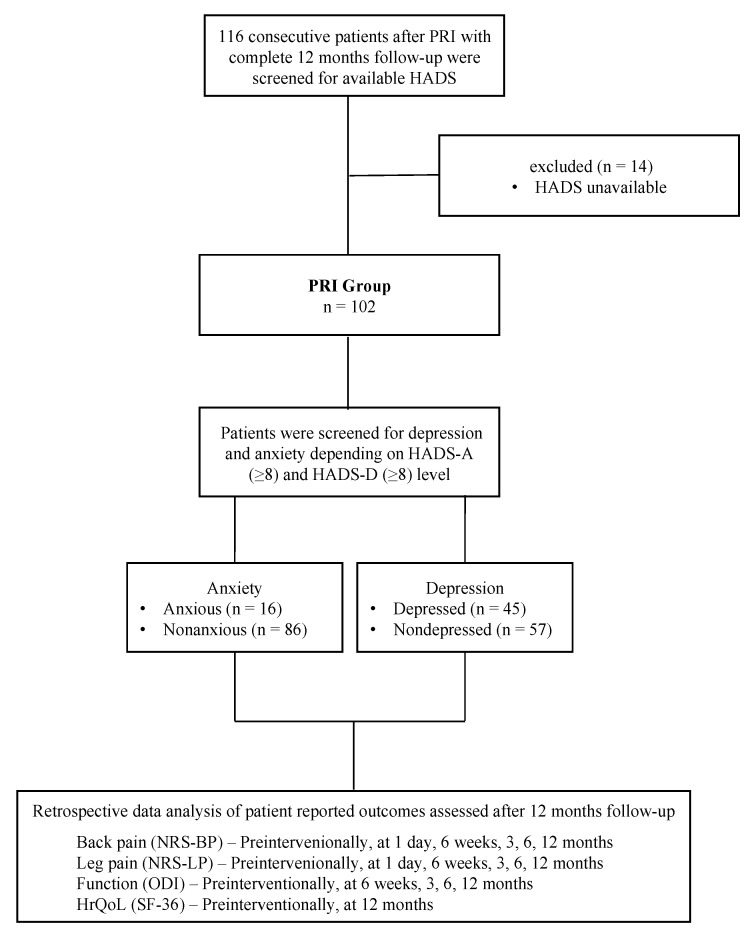
Flow chart. Schematic presentation of participant flow at the 12-month follow-up. PRI—periradicular infiltration therapy; HADS—Hospital Anxiety and Depression Scale; HrQoL—Health-related Quality of Life.

The present study was approved by the local ethics committee (2022-2852-Daten) and performed according to the Declaration of Helsinki 1975. All patients routinely signed informed consent for anonymously processing their data. Due to the retrospective design of the study, no study-specific consent form was signed. During the screening of electronic patient files, the selection criteria used were in the form of a fully completed Hospital Anxiety and Depression Scale (HADS) before the intervention. A flowchart of the patients included in the analysis is shown in Figure 1.

### 2.2. Intervention

**Figure 2:**
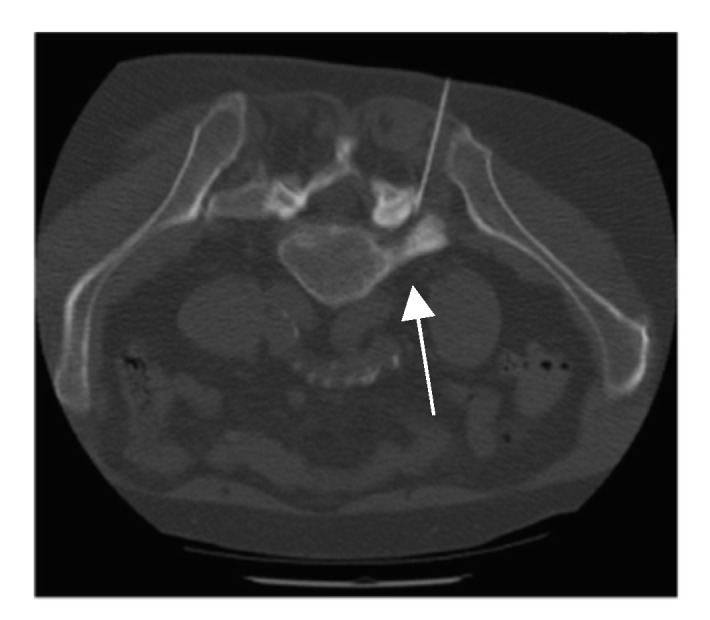
Computed tomography showing the positioning of the spinal needle lateral to the midline at the level of the affected nerve root via a transforaminal approach at the L5/S1 neuroforamen (white arrow).

Patients were treated under sterile conditions and placed in the prone position on the table of the CT scanner (BrightSpeed, GE Healthcare, Chicago, IL, USA). During PRI treatment, the needles were positioned lateral to the midline at the level of the affected nerve root via a transforaminal approach [17,18]. The medication was rinsed around the affected nerve root (Figure 2). The following medications were used during PRI: 1.5 mL local anesthetic (1% Xylocitin^®^, MIBE GmbH Arzneimittel, Sandersdorf-Brehna, Germany; Lidocaine hydrochloride) + 0.5 mL corticosteroid (Lipotalon^®^, Recordati Industria Chimica e Farmaceutica SpA, Milan, Italy; Dexamethasone). Before injection, needle aspiration was performed to prevent vascular spread. Due to CT-guided needle positioning, no contrast agent was used.

### 2.3. Clinical Scores

The scores presented below are summarized in Figure 1.

The patient’s back pain and leg pain perceptions were assessed using a numerical rating scale (NRS-BP and NRS-LP 0—no pain, 10—maximum pain). The German version of Oswestry Disability Index (ODI) was used to determine functional restriction. The ODI has become one of the most used measures of disability in back pain. A very high level of test–retest reliability was found (r = 0.91) in the literature. The total ODI score ranges from 0 (no disability) to 100 (maximum disability) [19,20]. The German version of the Health Short Form 36 (SF-36) was used to assess the condition of the health-related quality of life (HrQoL) [21] and included evaluation of the physical (pcs) and mental (mcs) total scores. The Short Form-36 (SF-36) is the most widely used health-related quality-of-life measure in research to date. The sum scales pcs and mcs allow the establishment of a scale value between 0 and 100. The subscale and sum values included represent a quantification of subjective health from the perception of the respondents. While a lower sum value correlates with a poorer quality of life, a higher sum value is associated with a better quality of life. Pain and Function Assessment (NRS-BP, NRS-LP, ODI) took place preprocedure and by telephone (except on first day with only NRS score) at the follow-up appointments at 6 weeks and at 3, 6, and 12 months. The preprocedure assessment and telephone assessment was conducted by an independent investigator not involved with clinical care. The SF-36 form was collected preprocedure and queried by mailing for the 12-month follow-up. To evaluate the influence of anxiety and depression on outcome after PRI, patients were retrospectively screened for a thoroughly answered hospital anxiety and depression scale (HADS) before the intervention. The HADS is an easy-to-use questionnaire. The HADS was found to perform well in assessing the symptom severity and caseness of anxiety disorders and depression in both somatic, psychiatric, and primary care patients, and in the general population. The Questionnaire consists of 14 questions that examine the symptoms of anxiety (HADS-A) with seven questions and the symptoms of depression (HADS-D) with seven questions [22]. Total scores between 0 and 7 indicate no abnormality; scores of 8 and above indicate anxiety or depression.Consequently, the patients were dichotomized into anxious and nonanxious groups and into depressed and nondepressed groups. To assess the therapeutic value of PRI, the improvements in the overall pain scale and the ODI compared to the preinterventional value (deltaNRSoverall and deltaODI) were compared to the minimal clinically important difference (MCID) for chronic complaints. According to previous work, the MCID was established for pain deltaNRSoverall = 2. For function, the deltaODI = 16% [23,24,25].

### 2.4. Data Analysis

To research the clinical improvement (concerning pain, function, and HRQoL) of patients with and without anxiety and depression, the subgroups’ patient-reported outcomes (PROs) with positive and negative HADS scores were compared (Figure 1). In the event of a missing HADS in the patient files, the case was excluded (listwise case exclusion).

### 2.5. Statistical Analysis

This work’s statistical evaluation was carried out using the software SPSS Statistics Version 28 for Macintosh (IBM, Armonk, NY, USA). The demographic data were checked using Student’s *t* test for independent samples, and the normal distribution of the data was assessed in advance using the Kolmogorov-Smirnov test. Categorical data were evaluated using Fisher’s exact test, and continuous data were evaluated using Student’s *t* test. Separate linear mixed models were built to estimate the effect of anxiety or depression on repeatedly measured PROs (NRS-BP, NRS-LP, ODI, SF-36) following PRI. The effects were adjusted for the respective baseline PRO and the timepoints of assessment. The two-sided level of significance was 0.05 for all statistical tests.

## 3. Results

### 3.1. Baseline Demographics

A total of 102 patients were available for data analysis with a complete 12-month follow-up and completely available HADS (Figure 1). The patient baseline demographics and clinical characteristics are listed in Table 1 and Table 2. Based on the HADS-A, the majority of patients who received a PRI were classified as nonanxious or nondepressed (84 patients (84%) were nonanxious; 57 (56%) were nondepressed). Considering the work status, there were no significant differences between the groups.

### 3.2. Influence of Anxiety and Depression on Baseline PROs

Most of the mean baseline PROs were significantly worse in anxious or depressed patients than in nonanxious or nondepressed patients (Table 2).

**Table 2:** Patient reported outcome scores at baseline before PRI in anxious and nonanxious and depressed and nondepressed patients.

	Total	Anxious	Non-Anxious	*p*-Value	Depressed	Nondepressed	*p*-Value
	Mean ± SD	Mean ± SD	Mean ± SD		Mean ± SD	Mean ± SD	
NRS-BP	5.8 ± 2.2	7.1 ± 1.9	5.5 ± 2.1	0.007	6.3 ± 2.1	5.4 ± 2.2	0.026
NRS-LP	7.1 ± 1.8	7.8 ± 1.4	6.9 ± 1.8	0.083	7.8 ± 1.1	6.5 ± 2.0	<0.001
ODI	47.3 ± 17.3	63.1 ± 13.0	44.3 ± 16.4	<0.001	58 ± 13.9	38.8 ± 14.7	<0.001
SF-36 (pcs)	33.0 ± 7.5	31.3 ± 7.8	33.3 ± 7.4	0.344	32.1 ± 7.4	33.6 ± 7.5	0.319
SF-36 (mcs)	47.8 ± 9.9	43.4 ± 10.9	48.7 ± 9.6	0.053	48.1 ± 10.7	47.6 ± 9.3	0.815

*p*-values from Fisher’s exact test; SD—single standard deviation. NRS-BP—numeric pain rating scale for back pain; NRS-LP—numeric pain rating scale for leg pain; ODI—Oswestry Disability Index to assess pain-related functional impairment; SF-36 pcs/mcs—Short From-36 physical sum scale/mental sum scale to assess health-related quality-of-life.

### 3.3. Influence of Anxiety and Depression on Clinical Improvement

**Figure 3:**
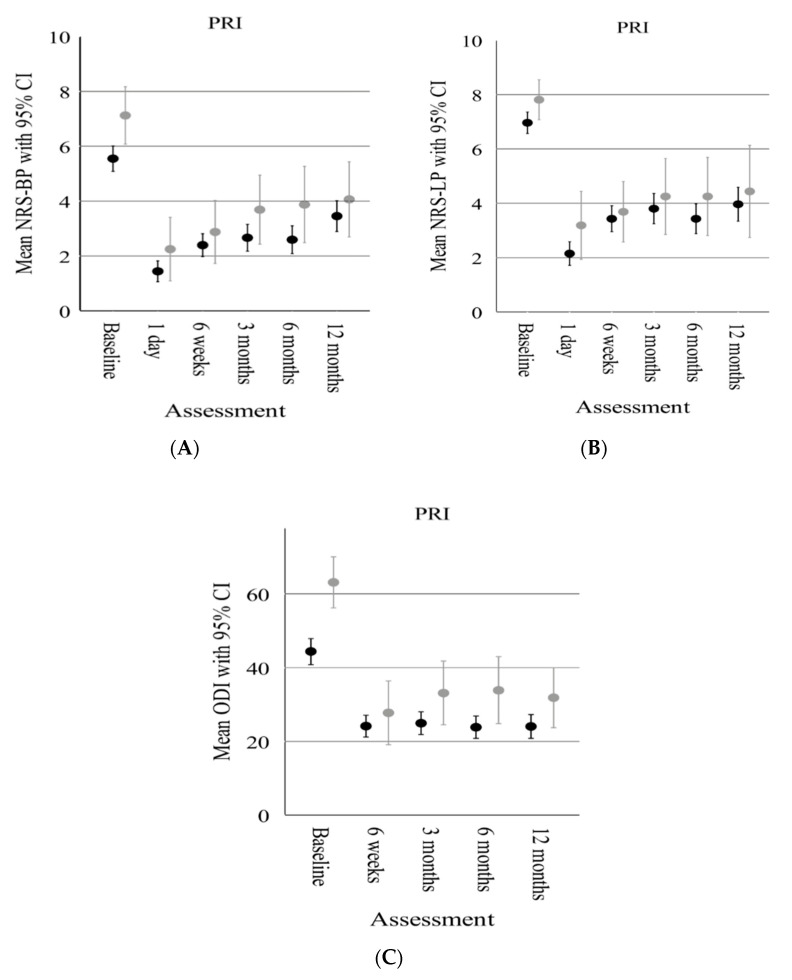
Comparison of the clinical outcome scores between anxious (grey dots and bars) and nonanxious (black dots and bars) patients following PRI over the follow-up period. (**A**) Comparison of mean NRS–Back Pain (BP). (**B**) Comparison of mean NRS–Leg Pain (LP). (**C**) Comparison of mean ODI. Whiskers represent the 95% confidence interval.

**Figure 4:**
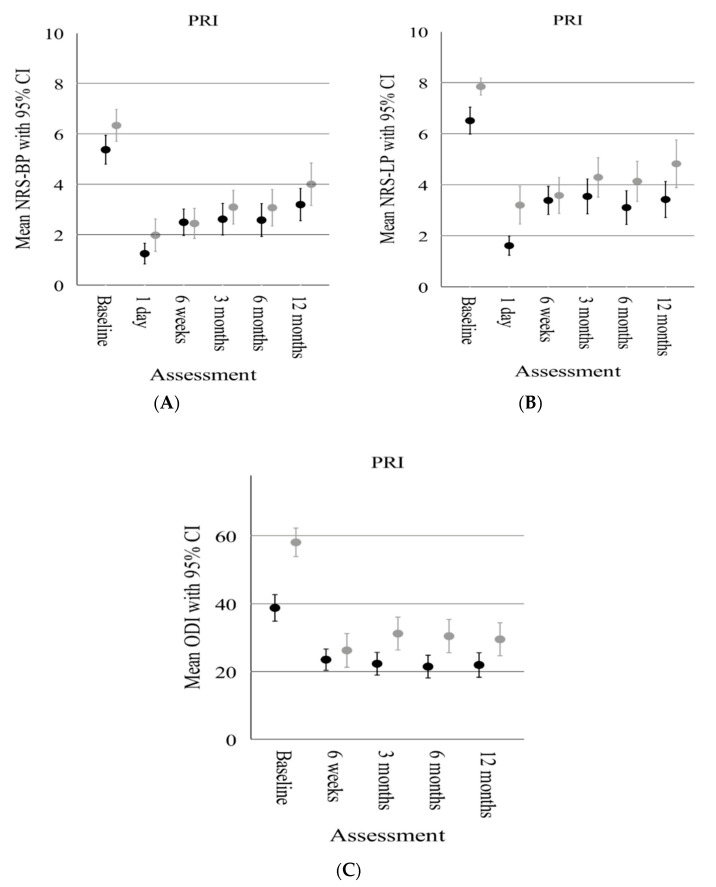
Comparison of the clinical outcome scores between depressed (grey dots and bars) and nondepressed (black dots and bars) patients following PRI over the follow-up period. (**A**) Comparison of mean NRS–Back Pain (BP). (**B**) Comparison of mean NRS–Leg Pain (LP). (**C**) Comparison of mean pain-related functional impairment (Oswestry Disablity Index—ODI). Whiskers represent the 95% confidence interval.

Regarding pain and functional improvement after PRI, both anxious and nonanxious patients, as well as depressed and nondepressed patients showed a reduction in pain (NRS-LP, NRS-BP) and functional improvement over a 12-month follow-up (Figure 3A–C and Figure 4A–C). In particular, anxious and depressed patients reported higher pain scores (NRS-BP, NRS-LP) and functional impairment than nonanxious and nondepressed patients during the observation period.

However, based on separate baseline PRO-adjusted linear mixed models, there was no significant difference in the estimated overall mean PRO improvement between anxious and nonanxious and depressed and nondepressed patients (Table 3 and Table 4).

### 3.4. Adverse Events

No differences between the groups were observed regarding the side effects after CT-based PRI. A total of 12 patients (12%) reported slightly transient and self-limiting side effects (1–4 h). These effects included numbness in the leg (eight patients); headache (two patients); and mild allergy, including redness of the face (two patients). No serious adverse events were reported during the 12-month observation period.

## 4. Discussion

The aim of the present study was to compare the outcome of patients after PRI with and without psychological confounders. Here, we report that anxious and nonanxious patients, as well as depressed and nondepressed patients showed a reduction in back pain and associated leg pain and functional improvement following CT-guided PRI over a 12-month follow-up. Regarding our primary hypothesis, we found no significant difference in the estimated overall mean PRO improvement between anxious or nonanxious and depressed or nondepressed patients. Even with MCID taken into account, the reduction in pain and improvement in function were clinically meaningful in both anxious and depressed patients. Conversely, anxiety and depression had a negative influence on pain and function at the baseline level. The subgroups also showed higher pain levels and functional impairment over the entire observation period. Our results underline the known fact that depression and anxiety can increase back pain and decrease functionality [26]. Furthermore, depression is associated with increased back pain and leg pain after spinal surgery and may result in less effective surgical outcomes [11,12]. Hence, pretreatment depression and anxiety may likely limit the effect of conservative treatment modalities such as lumbar infiltration therapies. However, the impact of psychological confounders on surgical therapies has not been studied in detail for conservative modalities such as PRI. According to our study, Kim et al. reported no significant difference in PRO after lumbar epidural steroid injections in patients with and without depression [14]. After one year of follow-up, the patients reported that pain, disability levels, and quality-of-life scores were worse in patients with depression. Precisely as in the present study, they highlighted that depression has a potentially negative effect on pain, disability, and HrQoL, but no significant impact on the treatment’s efficiency. Thus, the results for surgical therapy do not appear to be directly transferable to those for invasive pain management. Possible reasons for this could be higher complication rates of surgical therapy, the prolonged postoperative convalescence phase, or the postoperative pain experience. Furthermore, surgical therapy represents a considerable challenge, especially for patients with anxiety disorders. Contrary to our work, they focused on lumbar epidural steroid injections, including different approaches. However, it is known from the literature that other infiltration techniques may not have the same therapeutic value [5]. Our current study design included patients who underwent PRI in a standardized approach that is typically used in our clinical practice. Therefore, a direct comparison to the mentioned study is limited. Another recently published study conducted by Ozdemir et al. researched the effects of pretreatment depression and anxiety levels on transforaminal epidural steroid injections [15]. In contrast to our findings, they observed decreased benefits from the treatment as the depression scores increased. It is known that infiltration therapies such as PRI or transforaminal epidural steroid injections can reach a long-lasting effect of up to one year [5,27]. Consequently, the results reported by Ozdemir et al. may be affected by the limited follow-up period (3 months). Furthermore, in contrast to the present study, no psychosocial factors such as the work status, addictive disorders, or proven psychological pain of the patients studied were collected. However, it is known that such psychosocial factors have an impact on the health and well-being of individuals and, therefore, have an effect on LBP and the treatment outcome of LBP [12,13,28]. Hence, possible factors influencing the results of the above-mentioned study cannot be examined. In our study, there were no significant differences in work status between the groups examined and the findings are, therefore, comparable. Another major limitation of the previously mentioned study is a lack of a correct assignment of the patients based on their HADS level. The HADS is a valid instrument for the efficient, low-burden assessment of anxiety and depression in clinical trials with a low back pain population with known cutoff values [22]. Consequently, the dichotomization of the researched population is necessary (e.g., depressed/nondepressed, anxious/nonanxious). However, Ozdemir et al. dichotomized their patients based on treatment success (treatment success/no treatment success) and treated the HADS level as a metric variable in their statistics. Therefore, a study bias cannot be ruled out. Nevertheless, comparable to our findings, they did not see an effect of anxiety on the treatment outcome, but rather negatively affected preinjection functional scores. Among psychological confounders, depressive disorders are most commonly associated with chronic spinal conditions, with prevalence rates ranging from 56.2% to 80% [29]. In the present study, a correspondingly high prevalence of depression was found in patients undergoing PRI (49%). Furthermore, according to our findings, depression tends to increase the chance of developing low back pain and disability while interacting with physical symptoms [30]. In addition, depression has been shown to be associated with less successful treatment outcomes after surgery, partially after conservative therapy, and higher treatment drop-out rates among patients with chronic back pain [31]. Considering the increasing costs of treatments, including spinal injection therapies for chronic spinal disorders, modifiable patient-related factors (e.g., depression and anxiety) that can influence treatment success are becoming indispensable. Therefore, it is essential to accurately assess those psychological confounders in patients with chronic spinal disorders and then provide the appropriate management of depression and anxiety to achieve more successful therapeutic outcomes. Consequently, the reliable measurement of depression and anxiety is vital because it plays a crucial role in reaching treatment success in a population with chronic low back pain and associated radiculopathy.

Several limitations should be considered in interpreting our results. First, changes in psychological status after intervention were not evaluated. The present study focused on the effect of the preinterventional psychological status on the clinical outcome. Therefore, the psychological status after the intervention was not included. This status may change after interventional treatment. Furthermore, in the present study, the HADS was used as a screening method for the presence of anxiety and depression and not the possibly known diagnosis of anxiety disorder or depression of the patients concerned. Thus, we are not allowed to obtain information on a possibly already existing antidepressant or anxiolytic therapy. This must be considered when interpreting the results.

Second, we only collected the work status. However, other confounders that may affect postinterventional clinical results, such as social and educational background, were not recorded. However, it is known that such factors can influence the clinical outcome after lumbar fusion surgery [32,33], and may also affect the clinical outcome after lumbar infiltration therapies. Third, based on the retrospective study design, the present work included only a small number of anxious patients. Further research with an appropriate study design is needed for more valid comparability and generalizability. However, conclusions on the estimated effect of post-treatment outcomes by the presence of anxiety and depression are possible based on the mixed model that was applied in the presented study. Furthermore, the data collection by telephone may have an impact on the present results and must be considered when interpreting the results. Last, patients receiving PRI were included based on an imaged morphologically (MR-tomographically) secured single-level nerve root compression caused by a herniated disc, stenosis of the lateral recess, or neuroforamen. On the one hand, those pathologies reflect typical indications for PRI in everyday clinical practice. On the other hand, osseous and discogenic causes of nerve root compression may have different clinical responses to PRI and should be considered when interpreting the results [34]. In conclusion, the results of the present study represent a typical cohort in everyday clinical practice. However, considering the above-mentioned limitations, the generalization of the results has only limited scope. In the future, prospective studies that examine the influence of psychological and social factors on the treatment success of a lumbar infiltration therapy and thereby examine the influence of a possibly already existing therapy of these comorbidities are, therefore, imperative. Moreover, further research is needed to evaluate the influence of lumbar infiltration therapies on depression and anxiety changes. The study’s main strength is that this is the first study to examine the influence of anxiety and depression on the outcome after PRI over one year in a typical clinical practice. Furthermore, it should be emphasized that all included pathologies have been MR-tomographically validated.

## 5. Conclusions

The present study demonstrates that patients with depressive or anxious symptoms reported higher pain levels and more functional restrictions before and 12 months after PRI. However, patients with these confounders did show similar pain relief, functional improvement, or improvement of HrQoL. In conclusion, patients with chronic pain in degenerative lumbar disorders with accompanied depression or anxiety can expect to receive a similar gain in PRO compared to patients without depressive or anxious symptoms following PRI. In a general sense, if confirmed in a larger number of different approaches and clinical situations, it should be considered that depressed and anxious patients should not be excluded from the possibility of infiltrative interventions. Further, the outcome may be improved after identifying patients with psychosocial confounders by early education regarding the expected outcome, possibly early psychological intervention, and shifting the focus to multimodal therapy concepts.

## Figures and Tables

**Table 1:** Baseline demographic and clinical characteristics.

PRI	Total	Anxious	Nonanxious	*p*-Value	Depressed	Nondepressed	*p*-Value
	*n* = 102 (%)	16 (16%)	86 (84%)		45 (44%)	57 (56%)	
Sex: Male	45 (44%)	5 (31%)	40 (46%)	0.259	15 (33%)	30 (53%)	0.051 ^†^
Female	57 (56%)	11 (69%)	46 (54%)		30 (67%)	27 (47%)	
Age [yrs] (Mean ± SD)	64.1 ± 11.8	62.7 ± 16.1	64.3 ± 11.0	0.622	65.4 ± 12.2	63.1 ± 11.5	0.326 *
BMI [kg/m^2^] (Mean ± SD)	28.5 ± 5.0	29.5 ± 5.7	28.4 ± 4.8	0.418	29.1 ± 4.8	28.1 ± 5.1	0.297 *
HADS-A-level (Mean ± SD)	4.3 ± 3.2	9.9 ± 1.7	3.2 ± 2.2	<0.001	–	–	
HADS-D-level (Mean ± SD)	6.8 ± 3.9	–	–		10.5 ± 2.0	3.8 ± 2.1	<0.001 *

* *p*-values from Student’s *t*-test; † *p*-values from Fisher’s exact test; HADS—hospital anxiety and depression scale; SD—single standard deviation.

**Table 3:** Mean differences of post-treatment PRO according to anxiety at baseline estimated by linear mixed models.

	Post-Treatment PRO	Anxious	Nonanxious	Mean	(95% CI)	*p*-Value
		Estimated Overall Mean (SE)		Difference		
1	NRS-BP	2.7 (0.4) *	2.6 (0.1) *	0.1	(−0.7, 0.9)	0.757
1	NRS-LP	3.6 (0.4) *	3.4 (0.2) *	0.2	(−0.6, 1.0)	0.567
2	ODI	28.9 (3.0) ^†^	24.8 (1.2) ^†^	4.1	(−2.5, 10.7)	0.221
3	SF-36 (pcs)	37.6 (1.1)	35.8 (0.5)	1.8	(−0.7, 4,2)	0.155
3	SF-36 (mcs)	48.3 (0.4)	48.4 (1.0)	0.1	(−2.1, 2.3)	0.928

1 adjusted for baseline score and time point of assessment (1 day, 6 weeks, 3 months, 6 months, 12 months); 2 adjusted for baseline score and time point of assessment (6 weeks, 3 months, 6 months, 12 months); 3 adjusted for baseline score and time point of assessment (12 months); * indicates positive treatment effect concerning the minimal clinically important difference (MCID, DeltaNRS of 2); ^†^ indicates positive treatment effect concerning the minimal clinically important difference (MCID, DeltaODI of 16%); SE—standard error; CI—confidence interval; NRS-BP—numeric pain rating scale for back pain; NRS-LP—numeric pain rating scale for leg pain; ODI—Oswestry Disability Index to assess pain-related functional impairment; SF-36 pcs/mcs—Short From-36 physical sum scale/mental sum scale to assess health-related quality-of-life.

**Table 4:** Mean differences of post-treatment PRO according to depression at baseline estimated by linear mixed models.

	Post-Treatment PRO	Depressed	Nondepressed	Mean	(95% CI)	*p*-Value
		Estimated Overall Mean (SE)		Difference		
1	NRS-BP	2.7 (0.2) *	2.6 (0.2) *	0.1	(−0.5, 0.6)	0.863
1	NRS-LP	3.7 (0.2) *	3.2 (0.2) *	0.5	(−0.2, 1.1)	0.150
2	ODI	27.8 (1.9) ^†^	23.5 (1.6) ^†^	4.3	(−1.0, 9.7)	0.110
3	SF-36 (pcs)	36.1 (0.7)	36.1 (0.6)	0	(−1.9, 1.8)	0.942
3	SF-36 (mcs)	48.2 (0.6)	48.4 (0.5)	0.2	(−1.8, 1.3)	0.731

1 adjusted for baseline score and time point of assessment (1 day, 6 weeks, 3 months, 6 months, 12 months); 2 adjusted for baseline score and time point of assessment (6 weeks, 3 months, 6 months, 12 months); 3 adjusted for baseline score and time point of assessment (12 months); * indicates positive treatment effect concerning the minimal clinically important difference (MCID, DeltaNRS of 2); ^†^ indicates positive treatment effect concerning the minimal clinically important difference (MCID, DeltaODI of 16%); SE—standard error; CI—confidence interval; NRS-BP—numeric pain rating scale for back pain; NRS-LP—numeric pain rating scale for leg pain; ODI—Oswestry Disability Index to assess pain-related functional impairment; SF-36 pcs/mcs—Short From-36 physical sum scale/mental sum scale to assess health-related quality-of-life.

## Data Availability

Not applicable.
